# In vivo anatomical mapping of human locus coeruleus functional connectivity at 3 T MRI

**DOI:** 10.1002/hbm.24935

**Published:** 2020-01-28

**Authors:** Thomas Liebe, Jörn Kaufmann, Meng Li, Martin Skalej, Gerd Wagner, Martin Walter

**Affiliations:** ^1^ Clinical Affective Neuroimaging Laboratory Otto‐von‐Guericke University Magdeburg Germany; ^2^ Clinic for Neuroradiology Otto‐von‐Guericke University Magdeburg Germany; ^3^ Institute of Diagnostic and Interventional Radiology Jena University Hospital Jena Germany; ^4^ Department of Neurology Otto‐von‐Guericke University Magdeburg Germany; ^5^ Department of Psychiatry and Psychotherapy Jena University Hospital Jena Germany; ^6^ Department of Behavioral Neurology Leibniz Institute for Neurobiology Magdeburg Germany; ^7^ Department of Psychiatry and Psychotherapy Otto‐von‐Guericke University Magdeburg Germany; ^8^ Center of Behavioral Brain Sciences Otto‐von‐Guericke University Magdeburg Germany; ^9^ Department of Psychiatry Eberhard Karls University Tuebingen Tuebingen Germany

**Keywords:** locus coeruleus, neuromelanin sensitive MRI, resting‐state functional connectivity

## Abstract

The locus coeruleus (LC) is involved in numerous crucial brain functions and several disorders like depression and Alzheimer's disease. Recently, the LC resting‐state functional connectivity (rs‐fc) has been investigated in functional MRI by calculating the blood oxygen level–dependent (BOLD) response extracted using Montreal Neurological Institute (MNI) space masks. To corroborate these results, we aimed to investigate the LC rs‐fc at native space by improving the identification of the LC location using a neuromelanin sensitive sequence. Twenty‐five healthy male participants (mean age 24.8 ± 4.2) were examined in a Siemens MAGNETOM Prisma 3 T MRT applying a neuromelanin sensitive T1TSE sequence and functional MRI. We compared the rs‐fc of LC calculated by a MNI‐based approach with extraction of the BOLD signal at the exact individual location of the LC after applying CompCor and field map correction. As a measure of advance, a marked increase of regional homogeneity (ReHo) of time series within LC could be achieved with the subject‐specific approach. Furthermore, the methods differed in the rs‐fc to the right temporoparietal junction, which showed stronger connectivity to the LC in the MNI‐based method. Nevertheless, both methods comparably revealed LC rs‐fc to multiple brain regions including ACC, bilateral thalamus, and cerebellum. Our results are relevant for further research assessing and interpreting LC function, especially in patient populations examined at 3 T MRI.

## 1 INTRODUCTION

The locus coeruleus (LC) forms a bilateral brainstem nucleus located at the dorsal pons ventral of the fourth ventricle (Mouton, Pakkenberg, Gundersen, & Price, 1994). The importance of the LC for brain function becomes evident in the regulation of most of the norepinephrine (NE) exocytosis in the brain (Foote, Bloom, & Aston‐Jones, 1983; Morrison & Foote, 1986) and, along this line, the nucleus is involved in neurological disorders like dementia of the Alzheimer type (Tomlinson, Irving, & Blessed, 1981; Weinshenker, 2008; Zarow, Lyness, Mortimer, & Chui, 2003), Parkinson disease (German et al., 1992), anxiety disorder (McCall et al., 2015), and depression (Chan‐Palay & Asan, 1989; Weiss et al., 1994).

The foundation for the regulation of NE excretion by the LC is formed by its baseline activity, which regulates the response degree on sensory stimuli (Aston‐Jones & Cohen, 2005b). Aston‐Jones & Cohen (2005b) described the anterior cingulate cortex (ACC) as one of the relevant forebrain structures that send afferent connections to the LC and supervises its activity in terms of top‐down control. By conducting electrophysiological measurements in monkey brains, they found distinct activity states in the LC relevant for the amount of NE excretion to the extensive areas in the brain targeted by the LC (Aston‐Jones & Cohen, 2005a). The widespread efferent nerve fibers of the LC reach the neocortex, basal forebrain, limbic system, thalamus, hypothalamus, cerebellum and brainstem nuclei, where the NE spillover influences the local activity of the target nerve cells (Samuels & Szabadi, 2008a, 2008b). The LC‐NE system regulates diverse brain functions like attention, sensory perception, synaptic plasticity and memory or—in a perspective of performance—influences attentional shifts and adjustments of behavior (Samuels & Szabadi, 2008a; 2008b; Sara, 2009). For instance, in the so‐called “network reset” theory of Bouret and Sara (2005)), the authors suggest that the activation of LC‐NE system in response to a particular sensory event will produce or facilitate dynamic reorganization of neural networks which results in new functional networks regulating the adaptive behavioral output. The frontal lobe and the cingulate cortex have been shown to contain the highest density of noradrenergic (NA) fibers of all neocortical areas (Fuxe, Hamberger, & Hökfelt, 1968), which enable the modulation of cognitive flexibility and executive functioning of this brain network (Foote et al., 1983; Sara & Bouret, 2012).

When conducting LC functional connectivity (fc) measurements in MRI, we expect to elucidate its connectivity in accordance to previous concepts gained by anatomical surveys and electrophysiological animal studies. Recent human fMRI studies revealed the baseline functional connectivity of the LC (Murphy, O'Connell, O'Sullivan, Robertson, & Balsters, 2014; Jacobs, Müller‐Ehrenberg, Priovoulos, & Roebroeck, 2018; Wagner et al., 2018; Zhang, Hu, Chao, & Li, 2016). Furthermore, fc changes provoked by medication (Kline et al., 2016; Liebe et al., 2018; Song et al., 2017; Wagner, de la Cruz, Köhler, & Bär, 2017) and differences of the connectivity in Alzheimer's disease were found (Serra et al., 2018).

The limitation of previous research is that the identification of LC region of interest (ROI) and extraction of the blood oxygen level–dependent (BOLD) time series was based on Montreal Neurological Institute (MNI) space masks. The definition of LC was either based on averaging the LC location in a representative subject group measured with neuromelanin sensitive sequences (Keren, Lozar, Harris, Morgan, & Eckert, 2009) or by drawing the masks around MNI space coordinates derived from an anatomical atlas (Naidich et al., 2009). Besides the possibility of losing the exact intraindividual location of the LC in these analyses, coregistration of echo‐planar imaging (EPI) images to MNI space may lead to a shift of brainstem voxels and to misplaced EPI data inside the MNI space LC mask (Napadow, Dhond, Kennedy, Hui, & Makris, 2006).

In the present study, we therefore aim to systematically investigate the resting‐state functional connectivity of the LC by mapping the individual anatomical location of this cell group and to use this information for BOLD signal extraction. Specifically, we use the subject‐specific location of the LC revealed by a validated neuromelanin sensitive sequence (Keren et al., 2015; Sasaki et al., 2006) to extract the BOLD signal in the individual subject space and thereby intend to corroborate or conceivably improve rs‐fc measurement of the LC. We especially focus on comparing our results with previous LC measurements defining the LC location at group level or based on anatomical knowledge.

Furthermore, we calculated the regional homogeneity (ReHo) metric (Zang, Jiang, Lu, He, & Tian, 2004) of the voxel‐wise time series within the LC and hypothesized an increase in concordance of LC voxels identified with the subject‐specific method. To our knowledge, previous research was not approved by extraction of the BOLD signal from the exact individual localization of the LC outside of MNI space nor a comparison of this approach to the conventional procedure was published so far.

Finally, we present the optimized preprocessing options for LC measurements within a freely and commonly used fMRI data processing toolbox to assist and forward future research on this area.

## 2 METHODS

### 2.1 Participant characteristics

Twenty‐five male subjects (mean age 24.8 ± 4.2) were recruited by public advertisement. Inclusion criteria were defined by the absence of a current medical condition and history of major psychiatric illness as determined by medical history, physical examination, blood laboratory tests, electrocardiography, and toxicology findings. Potential healthy subjects who met the following criteria were excluded: Subjects with history of drug or alcohol dependency or abuse within the proceeding 6 months and subjects with serious unstable illness including hepatic, renal, gastroenterologic, respiratory, cardiovascular (including ischemic heart disease), endocrinologic, neurologic, immunologic, or hematologic disease, subjects with uncorrected hypothyroidism or hyperthyroidism and subjects with one or more seizures without a clear and resolved etiology. The criteria also excluded subjects with any illness likely to alter brain morphology and/or physiology like uncontrolled hypertension or diabetes and with the presence of any metallic (ferromagnetic) implants (heart pacemaker, aneurysm clips) or tattoos. The subjects were required to be off current medication for at least 6 weeks prior to inclusion. The study was approved by the institutional ethical review board of the University of Magdeburg, and all subjects gave written informed consent in accordance with the Declaration of Helsinki. The collected data of the study is available on request from the authors.

### 2.2 Image acquisition

Image acquisition was performed using a Siemens MAGNETOM Prisma 3 T MRI scanner with *syngo* MR E11 software and a 64‐channel head coil with the following parameters for the anatomical images: 3D‐MPRAGE sequence, echo time (TE) = 2.82 ms, repetition time (TR) = 2,500 ms, inversion time (T1) = 1,100 ms, flip angle = 7°, bandwidth = 140 Hz/pixel, acquisition volume = 256 × 256 × 192 mm^3^, isometric voxel size = 1.0 mm^3^, scan duration = 5 min 18 s.

Since neuromelanin acts as an endogenous MR contrast agent, that is, it shortens the longitudinal relaxation time T1, the LC appears hyperintense in high‐resolution T1‐weighted MRI at 3 T. Here a T1‐weighted TSE sequence according to the original work of Sasaki et al. (2006) with the following imaging parameters was applied: 14 axial slices, acquisition volume = 192 × 192 × 42 mm^3^, slice thickness = 2.50 mm, inter‐slice gap 0.5 mm, leading to an effective slice thickness of 3 mm, TR = 634.0 ms, TE = 10.0 ms, bandwidth = 165 Hz/Pixel, flip angle = 80°, scan duration = 10 min 50s.

Blood oxygenation level dependent (BOLD) signals were acquired using a multi‐band accelerated T2*‐weighted echo‐planar imaging (EPI) sequence provided by the Center for Magnetic Resonance Research of the University of Minnesota. We acquired eyes‐closed resting state fMRI data with the following parameters: 66 axial slices parallel to the anterior–posterior commissure plane covering the whole brain acquired in interleaved order, acquisition volume = 220 × 220 × 146 mm^3^, slice thickness = 2.20 mm, leading to a high resolution of 2.2 mm isotropic voxels and no gap, TR = 2000 ms, TE = 30.0 ms, flip angle = 150°, 320 volumes in total, scan duration = 11 min. We visually checked raw data for each dataset and each time point.

To be able to address the problem of geometric distortions in EPI caused by magnetic field inhomogeneities, a field map was acquired after the EPI sequence using a double‐echo gradient recalled echo (GRE) sequence (TE 1/2 = 4.92 ms/7.38 ms, TR = 660 ms, flip angle = 60°, isometric voxels size of 2.2 mm, FOV = 220 × 220 mm^2^, 66 slices aligned with the fMRI slices, scan duration = 2 min 13 s).

### 2.3 Definition of individual LC location

**Figure 1:**
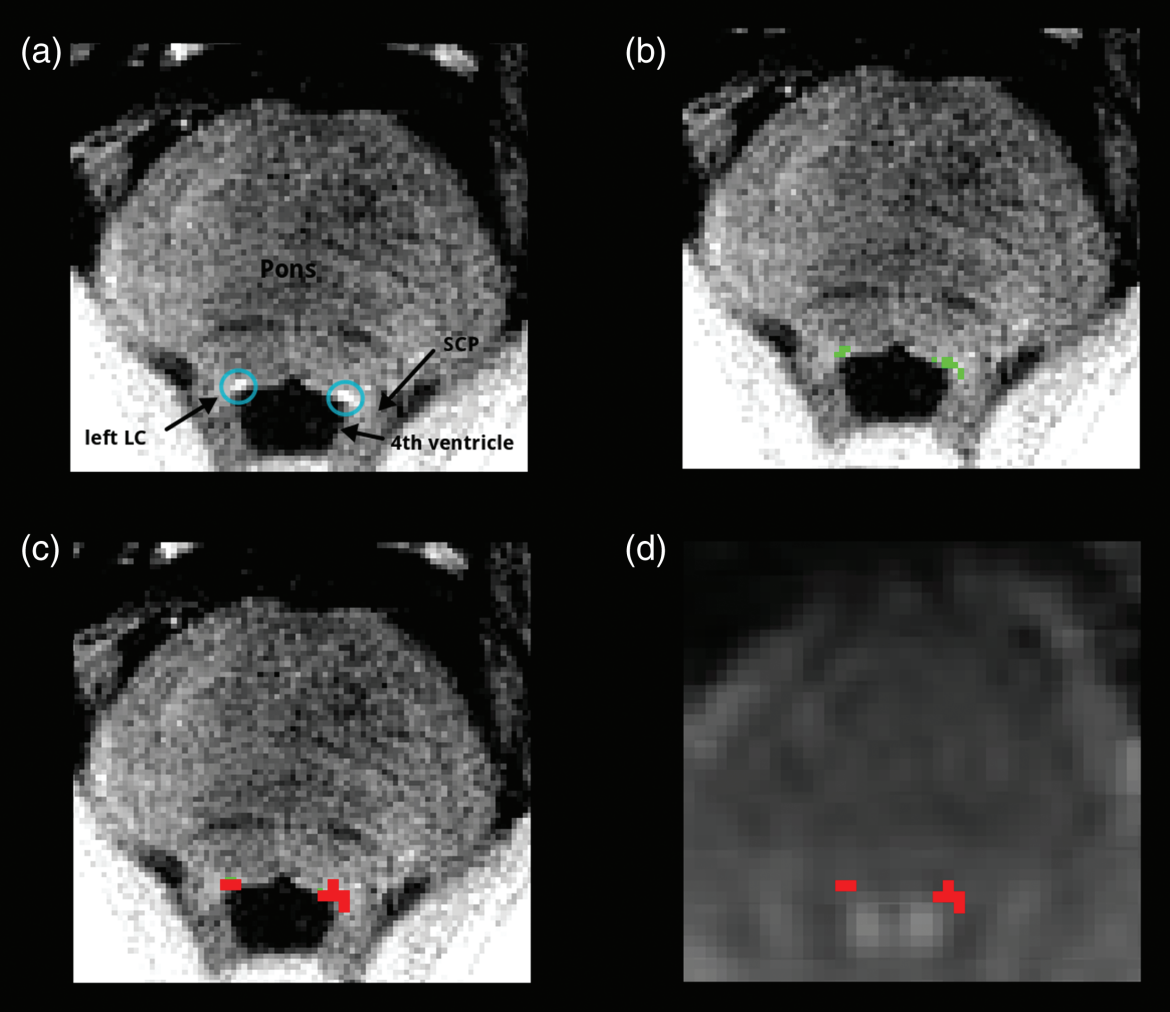
Raw T1TSE neuromelanin sensitive sequence (a). Individual masked LC (green) in T1TSE high‐resolution space (b). Mask (red) interpolated to BOLD resolution (c). Mask (red) in individual subject BOLD space (d). Anatomical structures marked in (a): left and right locus coeruleus (LC), pons, superior cerebellar peduncle (SCP), fourth ventricle

1. The LC location was manually drawn by a radiologist experienced in neuroradiology (3 years) in FSLview (https://fsl.fmrib.ox.ac.uk/fsl/) based on the neuromelanin contrast (Figure 1a).
2. T1TSE to T1MPRAGE: no coregistration was performed because of minimal movement of subjects and better results visually compared to automated coregistration methods (Data S1). FSL FLIRT was used to merge the matrix of the T1TSE sequence to T1MPRAGE space.
3. FSL FLIRT was used to interpolate the LC Mask in T1MPRAGE space with the resolution of functional space using the matrix gained in Step 2.
4. Finally, the obtained probability mask was binarized with fslmaths.

With this approach we minimized interpolation of the mask to only one step.

### 2.4 Definition of LC in MNI space mask

We used an LC mask which was created based on measurements of LC locations in 44 healthy subjects, which were registered to MNI space and surrounded by a mask containing LC peak signal coordinates observed at two SDs (Keren et al., 2009).

### 2.5 Functional MRI preprocessing

#### 2.5.1 Preprocessing based on MNI space mask

For the preprocessing of fMRI data, we used the standard preprocessing pipeline implemented in CONN (Whitfield‐Gabrieli & Nieto‐Castanon, 2012) with several modifications. The SPM12 (https://www.fil.ion.ucl.ac.uk/spm/) based preprocessing steps included simultaneous realignment, unwarp and field map correction of functional data, slice time correction (with slice timings provided to SPM because of the multiband sequence), ART based outlier detection (conservative settings with nuisance regression of time points exceeding the 95th percentile of movement compared to a normative sample to account for potential misplacement of LC, http://www.nitrc.org/projects/artifact_detect/), structural and functional direct segmentation and normalization to MNI space (structural target resolution 1 mm, functional target resolution 2 mm) and smoothing with a 4 mm kernel. Denoising included regression of white matter and CSF signals (CompCor, [Behzadi, Restom, Liau, & Liu, 2007]), removal of linear/quadratic trends, regression of subject motion (three‐rotation and three‐translation parameters as well as their first‐order temporal derivatives), removal of motion outliers (scrubbing) and band‐pass filtering at 0.008–0.09 Hz.

Then, the mean BOLD signal from the MNI space LC mask (Keren et al., 2009) was extracted within the coregistered, unsmoothed EPI images in MNI space and the timecourse was correlated to whole‐brain level.

#### 2.5.2 Preprocessing based on subject‐specific mask

A second preprocessing was conducted with the same parameters as described in Section 2.5.1 with the only exception that a direct functional‐to‐anatomical registration step was performed for every subject. Then, the mean BOLD signal was extracted from the unsmoothed EPI data coregistered to the individual anatomical image with the individual masks created as described in Section 2.3. In this way no translation of the subject‐specific masks into MNI space was necessary which may lead to a loss of location information. The LC timecourse was then correlated to the EPI dataset registered to MNI space (created in Section 2.5.1) to compare the results to the MNI space approach.

### 2.6 Functional MRI analysis

#### 2.6.1 Analysis of functional connectivity

For seed‐based correlation, Fisher‐transformed bivariate correlation coefficients of the extracted averaged time series within the LC masks and time series of MNI space whole brain voxels were calculated.

For ROI‐to‐ROI functional connectivity analysis, a matrix of Fisher‐transformed bivariate correlation coefficients between the LC masks' time series and MNI space whole‐brain ROI's was calculated. The target ROI's comprised the default atlas implemented in CONN covering whole brain (based on FSL Havard‐Oxford Atlas (https://fsl.fmrib.ox.ac.uk/fsl/fslwiki/) and cerebellar parcellation from AAL Atlas (Tzourio‐Mazoyer et al., 2002)). The complete list of ROI's included in the analysis is attached in Data S1.

The equations for calculation of seed‐to‐voxel and ROI‐to‐ROI connectivity used within CONN are presented in Data S1, too.

Cluster defining threshold was set to a *p* < .001 threshold at voxel level and only clusters below a threshold of FDR *p* < .05 were reported in the seed‐to‐voxel analysis. In the ROI‐to‐ROI analysis, connection‐level threshold was set to *p* < .05 and seed‐level threshold to an FDR *p* < .05 after permutation testing was performed (1,000 iterations) to account for the total number of connections included in the analysis, as suggested by the default CONN settings. For ROI‐to‐ROI analysis, *h* values, as a measure of effect size, were reported, which represent the mean Fischer transformed pairwise correlations between LC and the connected ROI's (Tables 2 and 3).

Finally, the LCrs‐fc z‐transformed correlation maps in the MNI space of the two datasets were compared regarding both the ROI‐to‐ROI functional connectivity analysis and the seed‐to‐voxel approach in a second level general linear model analysis using two‐sided‐paired *t* tests (Whitfield‐Gabrieli & Nieto‐Castanon, 2012).

#### 2.6.2 Analysis of regional homogeneity (ReHo)

In fc analyses, time courses are averaged over the chosen region of interest, and no information is provided about the homogeneity of the data *within* the cluster. Therefore, we calculated the ReHo metric (Zang et al., 2004) of the voxel‐wise time series within the LC masks, which is computed by the Kendall coefficient of concordance (KKC) of the BOLD time‐series of neighboring voxels. ReHo KCC (Zang et al., 2004) is a nonparametric statistical approach with values range from 0 (no agreement) to 1 (perfect agreement of time series). Higher values in ReHo indicate a greater similarity among the time series of neighboring voxels.

ReHo was calculated with BRAinNetome fMRI Toolkit (BRANT [Xu, Liu, Zhan, Ren, & Jiang, 2018]). The equation for calculation of ReHo KKC within BRANT is detailed in Data S1. For the MNI space approach, the MNI space mask was used to extract the BOLD time series from the unsmoothed EPI data from each voxel within the mask (in accordance to Section 2.5.1). For the subject‐specific approach, BOLD time series were extracted from EPI images coregistered to the individual subject anatomical scan within the subject‐specific LC masks (in accordance to Section 2.5.2). ReHo was calculated with a neighbor type of 26 voxels without smoothing. No voxels outside the masks were included in the analysis to avoid bias from non‐LC related time courses. The Kendall's coefficient of concordance (KCC) of each mask was averaged with fslstats. MNI and subject‐specific KKC values were compared with a Wilcoxon signed‐rank test.

## 3 RESULTS

### 3.1 Characterization of MNI and subject‐specific LC masks

The mean number of voxels contained in the subject‐specific masks was comparable to the MNI space mask (subject‐specific masks 48.28 ± 17.42 voxels, MNI space mask 50 voxels).

The smallest subject‐specific mask contained 15 voxels, the biggest a total of 95 voxels. The mode (50 voxels) of the subject‐specific masks exactly met the voxel size of the MNI space mask.

### 3.2 ReHo

We found significant greater ReHo KCC values in the subject‐specific method compared to the MNI space approach (*p* < .001). All 25 subjects clearly showed higher concordance of time series within LC after extraction of the signal at the subject‐specific location. Table 1 shows mean LC ReHo values of all subjects.

**Table 1:** Mean Kendall's coefficients of concordance (KCC) within the LC masks in comparison of subject‐specific and MNI space approach from all subjects with greater agreement of time series in the subject‐specific approach (*p* < .001)

Subject ID	Mean KCC MNI mask	Mean KCC individual mask
1	0.39	0.59
2	0.42	0.66
3	0.43	0.62
4	0.44	0.62
5	0.41	0.64
6	0.44	0.66
7	0.39	0.61
8	0.43	0.63
9	0.41	0.59
10	0.42	0.66
11	0.46	0.62
12	0.45	0.60
13	0.42	0.61
14	0.39	0.65
15	0.45	0.66
16	0.38	0.58
17	0.38	0.63
18	0.45	0.64
19	0.46	0.59
20	0.42	0.64
21	0.41	0.62
22	0.42	0.63
23	0.39	0.64
24	0.40	0.65
25	0.41	0.65

### 3.3 ROI‐to‐ROI rs‐fc of LC to all the brain

#### 3.3.1 Anatomical location approach

**Figure 2:**
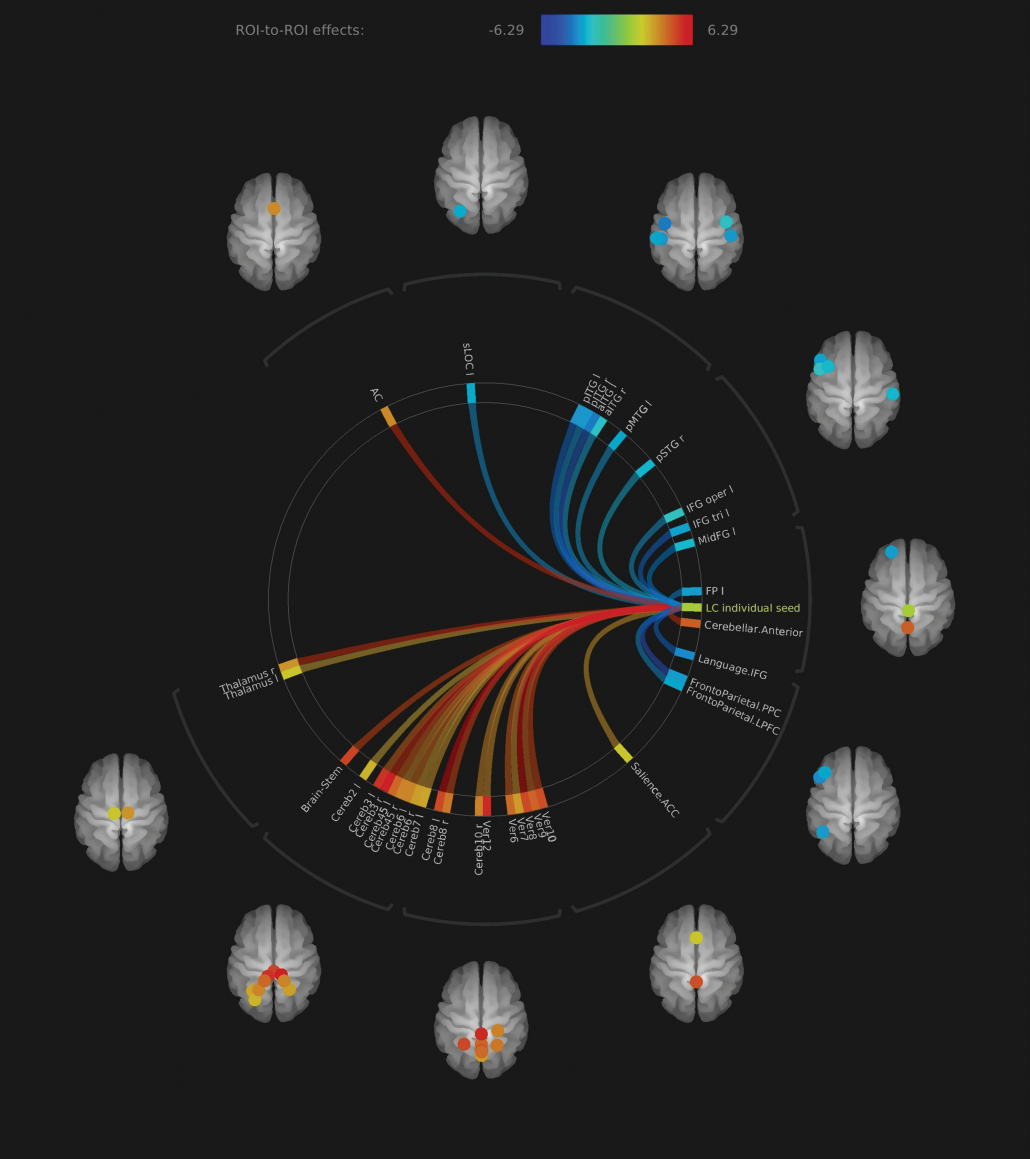
Rs‐fc of LC to all the brain in ROI‐to‐ROI analysis, individual seed anatomical location approach. Significant positive functional connectivity was found to bilateral thalamus, anterior cingulate cortex and cerebellum. Negative functional connectivity was found to (fronto)temporal regions (all presented regions FDR *p* < .05)

By extracting the BOLD time series out of the individual subject LC location, we found significant positive ROI‐to‐ROI LC rs‐fc including bilateral thalamus (right thalamus FDR *p* < .001, left thalamus FDR *p* < .006), ACC (FDR *p* < .001) and cerebellar regions. Furthermore, the LC exhibited negative baseline connectivity to frontotemporal regions. A detailed description of significant connections is presented in Figures 2 and 4, and Table 2.

**Table 2:** Rs‐fc of LC to whole‐brain ROI's in individual seed approach

Analysis unit	Statistic	p‐FDR	*h* values
Seed LC individual	F(5)(20) = 3.24		
	Intensity = 152.94	0.0000	
	Size = 37	0.0000	
LC individual‐Cereb8 l	T(24) = 6.29	0.0000	0.11954
LC individual‐Ver8	T(24) = 6.13	0.0000	0.11969
LC individual‐Cereb3 r	T(24) = 5.60	0.0000	0.13168
LC individual‐AC	T(24) = 5.58	0.0000	0.08493
LC individual‐Thalamus r	T(24) = 5.45	0.0000	0.08039
LC individual‐networks.cerebellar.anterior	T(24) = 5.36	0.0000	0.10805
LC individual‐Ver10	T(24) = 5.04	0.0000	0.11597
LC individual‐Brain‐Stem	T(24) = 5.02	0.0000	0.12235
LC individual‐Cereb8 r	T(24) = 4.91	0.0001	0.09465
LC individual‐networks.FrontoParietal.PCCl	T(24) = −4.88	0.0001	−0.07647
LC individual‐Cereb45 l	T(24) = 4.55	0.0001	0.10263
LC individual‐Ver6	T(24) = 4.55	0.0001	0.10116
LC individual‐aITG l	T(24) = −4.48	0.0002	−0.09244
LC individual‐Cereb3 l	T(24) = 4.42	0.0002	0.13168
LC individual‐Ver9	T(24) = 4.33	0.0002	0.10562
LC individual‐Language.IFG	T(24) = −4.20	0.0003	−0.05467
LC individual‐Cereb6 l	T(24) = 4.19	0.0003	0.08967
LC individual‐pITG l	T(24) = −4.18	0.0003	−0.07949
LC individual‐IFG tri l	T(24) = −4.18	0.0003	−0.07202
LC individual‐MidFG l	T(24) = −3.73	0.0010	−0.06161
LC individual‐Cereb10 r	T(24) = 3.71	0.0011	0.08988
LC individual‐Cereb45 r	T(24) = 3.68	0.0012	0.08865
LC individual‐FP l	T(24) = −3.56	0.0016	−0.07501
LC individual‐Ver12	T(24) = 3.46	0.0020	0.13614
LC individual‐networks.FrontoParietal.LPFCl	T(24) = −3.42	0.0022	−0.06878
LC individual‐Ver7	T(24) = 3.40	0.0024	0.07627
LC individual‐Cereb7 l	T(24) = 3.38	0.0025	0.07292
LC individual‐sLOC l	T(24) = −3.36	0.0026	−0.06776
LC individual‐pITG r	T(24) = −3.35	0.0027	−0.07805
LC individual‐Salience.ACC	T(24) = 3.29	0.0031	0.054624
LC individual‐Cereb6 r	T(24) = 3.21	0.0037	0.07436
LC individual‐IFG oper l	T(24) = −3.19	0.0039	−0.04988
LC individual‐pMTG l	T(24) = −3.19	0.0039	−0.06912
LC individual‐Thalamus l	T(24) = 2.98	0.0066	0.05483
LC individual‐aITG r	T(24) = −2.96	0.0068	−0.05315
LC individual‐Cereb2 l	T(24) = 2.94	0.0071	0.06539
LC individual‐pSTG r	T(24) = −2.81	0.0098	−0.06227

#### 3.3.2 MNI based approach

**Figure 3:**
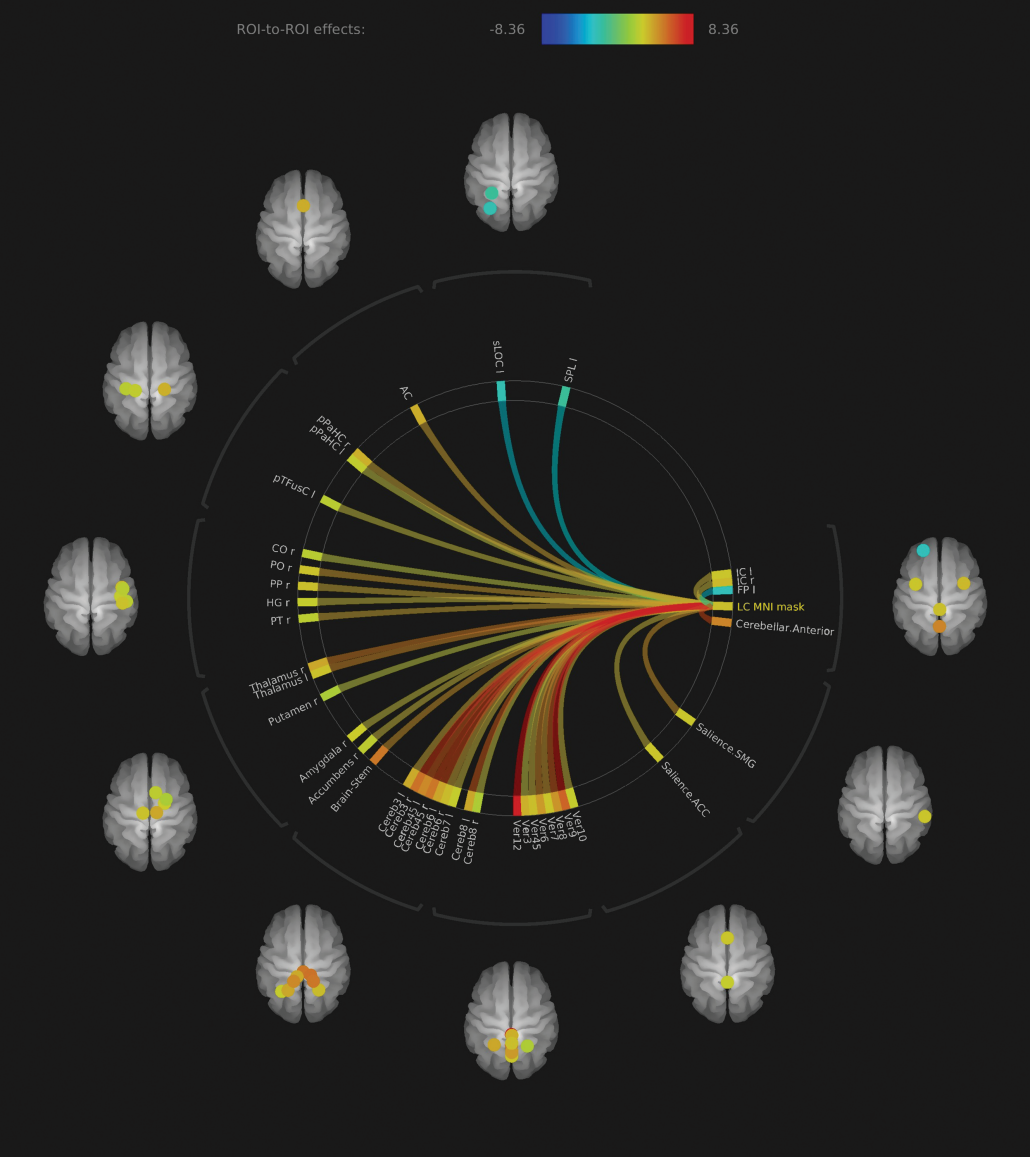
Rs‐fc of LC to all the brain in ROI‐to‐ROI analysis, MNI space mask approach. Significant functional connectivity to bilateral thalamus, anterior cingulate cortex and cerebellum. Compared to the individual seed approach, the connectivity to some regions was more significant including amygdala and nucleus accumbens without reaching statistical significant difference in direct comparison (all presented regions FDR *p* < .05)

**Figure 4:**
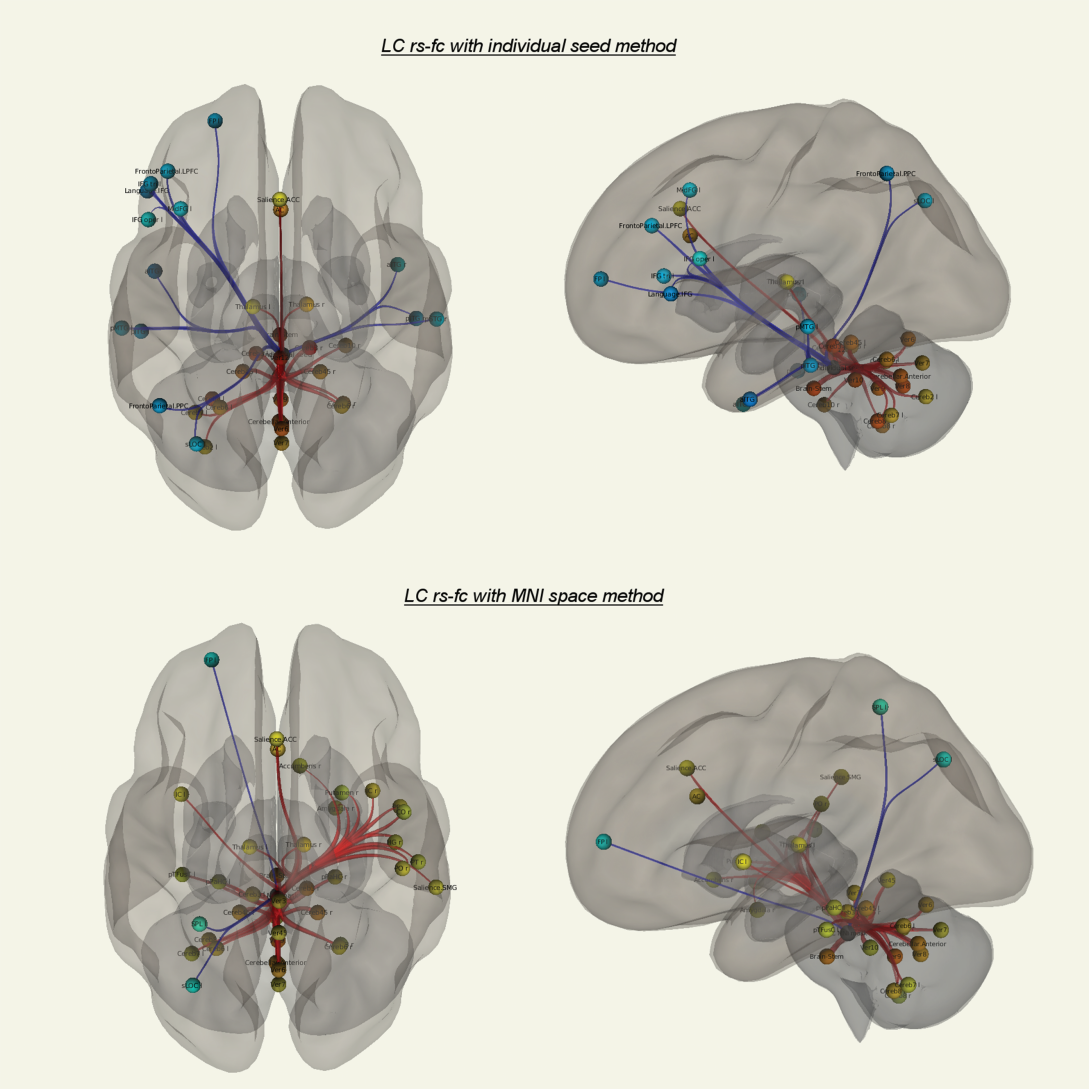
ROI's with significant LC rs‐fc in the individual seed (top row) and MNI space (bottom row) method. Red: positive connectivity, blue: negative connectivity (all presented regions FDR *p* < .05). For detailed significance values see Tables 3 and 4. No significant differences of both approaches in ROI‐to‐ROI analysis in direct comparison, although differences in connectivity significance to some frontotemporoparietal regions are visible. Similar strong rs‐fc of LC to ACC, bilateral thalamus, and bilateral cerebellum in both approaches

By extracting the BOLD time series based on the MNI space mask, we also found significant positive baseline ROI‐to‐ROI LC rs‐fc connectivity including bilateral thalamus (FDR *p* < .001), anterior cingulate cortex (FDR *p* < .001) and cerebellar regions. Furthermore, the LC exhibited positive baseline connectivity to the basal ganglia, the right amygdala (FDR *p* < .004) and right nucleus accumbens (FDR *p* < .003). For a detailed description of significant connections, see Figures 3 and 4 and Table 3.

**Table 3:** Rs‐fc of LC to whole‐brain ROI's in MNI seed approach

Analysis unit	Statistic	p‐FDR	*h* values
Seed LC MNI mask	F(5)(20) = 2.83		
	Intensity = 174.33	0.0000	
	Size = 40	0.0000	
LC MNI mask‐Ver12	T(24) = 8.36	0.0000	0.20944
LC MNI mask‐Ver9	T(24) = 8.02	0.0000	0.14149
LC MNI mask‐Cereb3 r	T(24) = 7.11	0.0000	0.13282
LC MNI mask‐Cereb45 r	T(24) = 6.75	0.0000	0.12826
LC MNI mask‐Cereb6 l	T(24) = 6.66	0.0000	0.09611
LC MNI mask‐networks.cerebellar.anterior	T(24) = 6.64	0.0000	0.12000
LC MNI mask‐Cereb45 l	T(24) = 6.11	0.0000	0.11598
LC MNI mask‐Ver8	T(24) = 6.10	0.0000	0.10862
LC MNI mask‐Cereb8 l	T(24) = 6.10	0.0000	0.09540
LC MNI mask‐Thalamus r	T(24) = 5.11	0.0000	0.09553
LC MNI mask‐Ver6	T(24) = 4.91	0.0001	0.10498
LC MNI mask‐Thalamus l	T(24) = 4.73	0.0001	0.07692
LC MNI mask‐Brain‐Stem	T(24) = 4.43	0.0002	0.12924
LC MNI mask‐networks.salience.SMG	G T(24) = 4.41	0.0002	0.02325
LC MNI mask‐Ver7	T(24) = 4.30	0.0002	0.07474
LC MNI mask‐PO r	T(24) = 4.18	0.0003	0.07544
LC MNI mask‐ACC	T(24) = 4.00	0.0005	0.09175
LC MNI mask‐Cereb6 r	T(24) = 3.98	0.0005	0.07977
LC MNI mask‐pPaHC r	T(24) = 3.96	0.0006	0.08969
LC MNI mask‐Cereb3 l	T(24) = 3.91	0.0007	0.09428
LC MNI mask‐PT r	T(24) = 3.86	0.0008	0.05954
LC MNI mask‐PP r	T(24) = 3.77	0.0009	0.07773
LC MNI mask‐Ver45	T(24) = 3.55	0.0016	0.07858
LC MNI mask‐IC l	T(24) = 3.50	0.0018	0.07376
LC MNI mask‐Ver10	T(24) = 3.44	0.0021	0.06618
LC MNI mask‐Cereb7 l	T(24) = 3.40	0.0023	0.06449
LC MNI mask‐Salience.AC	C T(24) = 3.38	0.0025	0.07410
LC MNI mask‐Accumbens r	T(24) = 3.37	0.0025	0.06013
LC MNI mask‐IC r	T(24) = 3.32	0.0029	0.08315
LC MNI mask‐HG r	T(24) = 3.25	0.0034	0.06545
LC MNI mask‐FP l	T(24) = −3.23	0.0035	−0.06308
LC MNI mask‐pTFusC l	T(24) = 3.20	0.0038	0.05595
LC MNI mask‐Amygdala r	T(24) = 3.18	0.0040	0.06964
LC MNI mask‐Cereb8 r	T(24) = 3.03	0.0058	0.04949
LC MNI mask‐sLOC l	T(24) = −2.98	0.0065	−0.05743
LC MNI mask‐Ver3	T(24) = 2.91	0.0077	0.08993
LC MNI mask‐pPaHC l	T(24) = 2.91	0.0077	0.06267
LC MNI mask‐Putamen r	T(24) = 2.77	0.0106	0.04810
LC MNI mask‐CO r	T(24) = 2.75	0.0112	0.05649
LC MNI mask‐SPL l	T(24) = −2.75	0.0113	−0.03985

#### 3.3.3 Visual comparison of both methods

In the ROI‐to‐ROI analysis, we found no significant differences between both methods in direct comparison. Nevertheless, some regions differed in effect size to a certain extent, for example, left temporal regions in the MNI masked method with positive connectivity and bilateral regions in the subject‐specific method with negative connectivity. The axes ACC–LC, LC–bilateral thalamus, and LC–cerebellum show similar effect sizes in both methods (Figure 4, Tables 2 and 3).

### 3.4 Seed‐to‐voxel rs‐fc LC to all the brain

#### 3.4.1 Anatomical location approach

**Figure 5:**
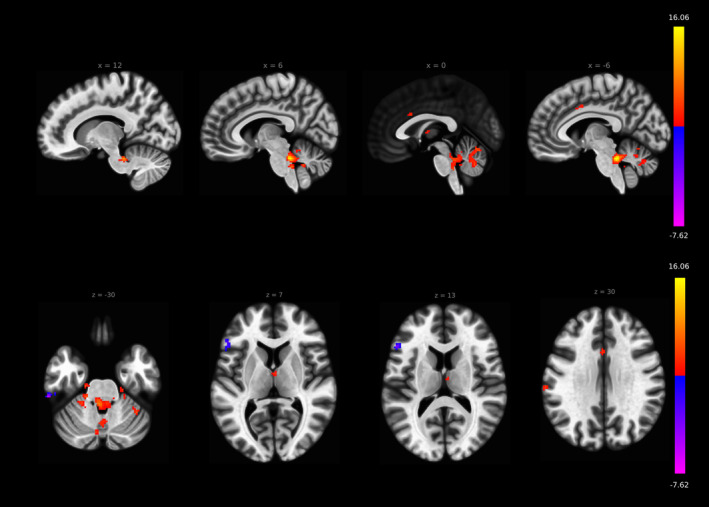
LC rs‐fc in the seed‐to‐voxel analysis reveals significant connectivity to ACC (FDR *p* < .026, cluster size 20), bilateral thalamus (FDR *p* < .01, cluster size 25), and cerebellum (FDR *p* < .001, cluster size 199) in the individual subject seed approach

In the seed‐to‐voxel analysis, LC rs‐fc revealed significant positive connectivity to ACC (FDR *p* < .026, cluster size 20), bilateral thalamus (FDR *p* < .01, cluster size 25) and cerebellum (FDR *p* < .001, cluster size 199) in the individual subject seed approach (Figure 5). Furthermore, the parahippocampal gyrus was positively connected to the LC (right parahippocampal gyrus FDR *p* < .003, cluster size 11; left parahippocampal gyrus *p* < .004, cluster size 13). Negative LC rs‐fc was shown to frontotemporal neocortical areas like left frontal pole, left superior, middle and inferior frontal gyrus and left lateral occipital cortex (Table 4).

**Table 4:** LC rs‐fc in the seed‐to‐voxel analysis in the individual subject seed approach

*x*	*y*	*z*	Size	Size p‐FDR
−6	−38	−26	420	0.000000
−2	−66	−18	199	0.000000
−42	54	−12	169	0.000000
−52	26	12	122	0.000000
−62	−30	−26	109	0.000000
−36	22	50	95	0.000002
−66	−24	28	66	0.000059
−12	−34	−16	49	0.000568
−44	−8	−34	47	0.000681
−28	−78	50	44	0.000968
22	−24	−26	36	0.003138
−14	−50	−18	34	0.003701
−24	−40	−30	34	0.003701
−18	−20	−28	32	0.004822
20	0	72	27	0.010845
−18	−32	−30	26	0.012199
2	−8	10	25	0.013809
−50	−62	44	24	0.014896
30	−36	−26	24	0.014896
−40	4	−14	22	0.018851
38	−50	−30	22	0.018851
32	−40	−44	22	0.018851
−8	6	38	20	0.025622
36	12	−16	20	0.025622
−46	38	−12	19	0.030098
−2	16	28	18	0.033000
16	−30	−10	18	0.033000
−14	38	−20	18	0.033000

#### 3.4.2 MNI‐based approach

In the MNI‐based method, LC rs‐fc revealed significant connectivity to ACC (FDR *p* < .001, cluster size 48), bilateral thalamus (left thalamus FDR *p* < .001, cluster size 60; right thalamus *p* < .01 cluster size 24), bilateral cerebellum (FDR *p* < .001, cluster size 1,120) and right TPJ (FDR *p* < .001, cluster size 77). Negative connectivity was shown to left lateral occipital cortex and left frontal pole (Table 5).

**Table 5:** LC rs‐fc in the seed‐to‐voxel analysis in the MNI space mask approach

*x*	*y*	*z*	Size	Size p‐FDR
8	−38	−32	1,120	0.000000
−32	−58	56	86	0.000009
62	−28	28	77	0.000019
−14	−20	6	60	0.000117
−36	−48	−32	60	0.000117
2	26	26	48	0.000579
42	6	−6	39	0.002083
34	−48	−24	31	0.007186
−12	−52	−30	30	0.007642
36	10	−16	28	0.009005
−22	−30	−28	28	0.009005
−30	−38	−32	27	0.009939
−44	−38	−24	26	0.011071
14	−12	6	24	0.015082
−24	30	−12	23	0.017119
−24	50	30	22	0.018421
−6	−70	−28	22	0.018421
−10	−48	−46	21	0.021282
18	−4	−14	20	0.024743
14	−28	−12	19	0.028946
2	−8	8	18	0.034073
−38	12	−14	17	0.040360
−22	−72	58	16	0.048110

#### 3.4.3 Differences between seed‐to‐voxel anatomical and MNI based approach

**Figure 6:**
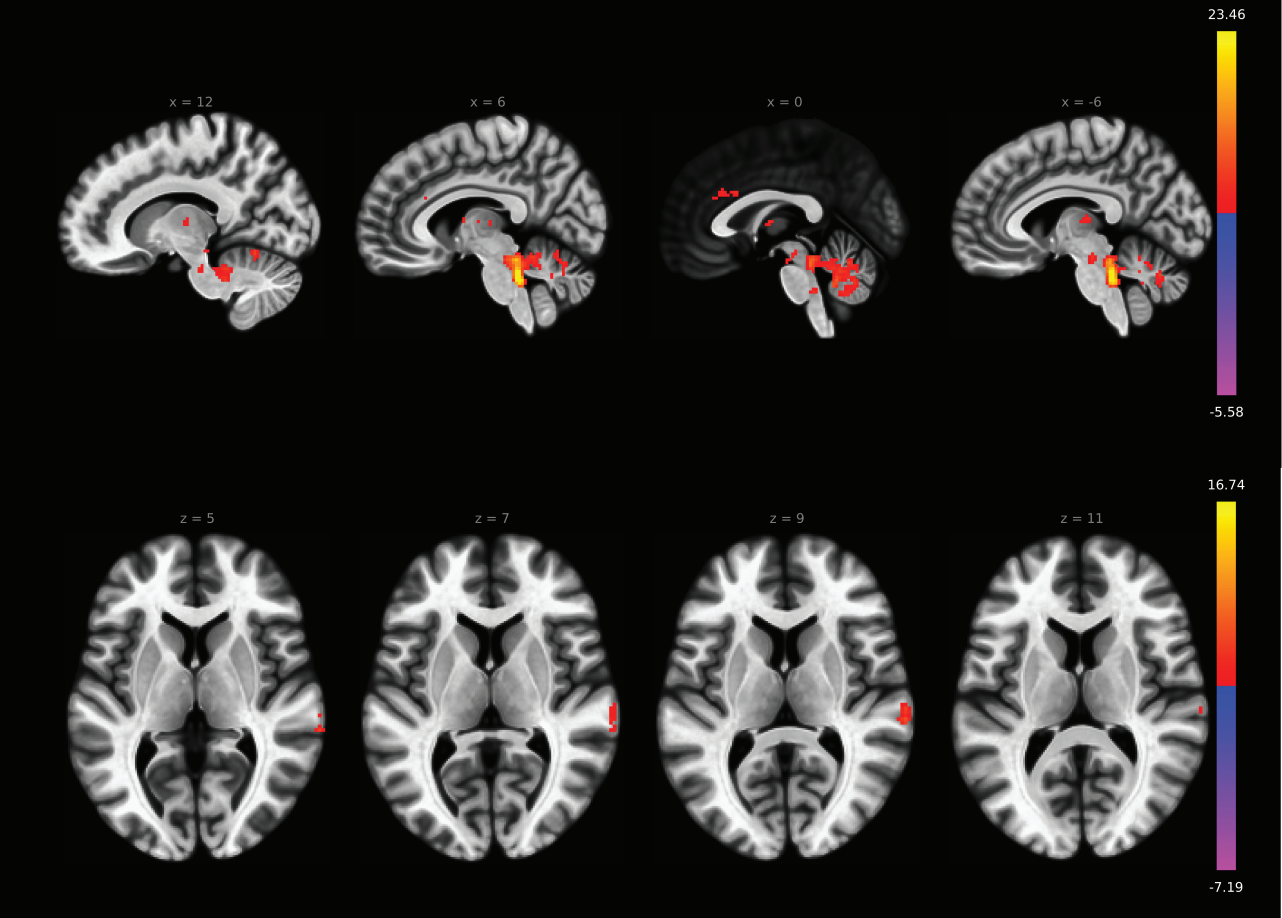
LC rs‐fc in the seed‐to‐voxel analysis reveals significant connectivity to ACC (FDR *p* < .001, cluster size 48), bilateral thalamus (left thalamus FDR *p* < .001, cluster size 60; right thalamus *p* < .01, cluster size 24), bilateral cerebellum (FDR *p* < .001, cluster size 1,120), and right TPJ (FDR *p* < 0.001, cluster size 77) in the MNI space mask approach

**Figure 7:**
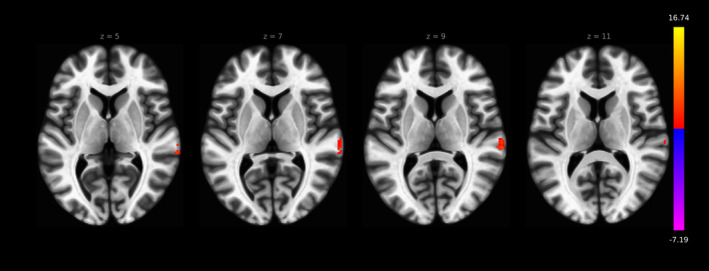
LC rs‐fc in the seed‐to‐voxel analysis differs between MNI seed–based and individual seed–based method: the right TPJ showed higher rs‐fc to LC in the MNI seed–based method (FDR *p* < .001, cluster size 47)

The seed‐to‐voxel LC rs‐fc analysis revealed the axes ACC–LC (subject‐specific mask: FDR *p* < .026, cluster size 20; MNI mask: FDR *p* < .001, cluster size 48), LC‐bilateral thalamus (subject‐specific mask: FDR *p* < .01, cluster size 25; MNI mask: left thalamus FDR *p* < .001, cluster size 60, right thalamus *p* < .01, cluster size 24) and LC‐cerebellum (subject‐specific mask: FDR *p* < .001, cluster size 199; MNI mask: FDR *p* < .001, cluster size 1,120) in both MNI and subject‐specific methods and they are in accordance to the ROI‐to‐ROI analyses presented before (Figures 5 and 6, Tables 4 and 5). Significant differences were found with stronger LC rs‐fc to right TPJ in the MNI space mask approach (FDR *p* < .001, cluster size 47, Figure 7).

## 4 DISCUSSION

In agreement with previous studies, we found significant rs‐fc of the LC to ACC, bilateral thalamus and bilateral cerebellum with both MNI‐based and subject‐specific approaches and only the right TPJ showed significant differences with stronger LC connectivity in the MNI mask based method. Nevertheless, we could reveal a clear improvement of extraction of relevant LC BOLD signal with the subject‐specific approach shown by increased concordance in terms of regional homogeneity of the extracted time series.

The mean and median size of the subject‐specific masks nearly or exactly met the size of the MNI space mask. The MNI space mask is based on the coordinates of the LC signal peak location of 44 healthy subjects observed at two SDs from the sample mean. The similarity of the results shows that replication of LC delineation leads to comparable sizes of a mean LC mask.

On the other site, the SD of the subject‐specific masks created in this study was rather high, reflecting the individual variability of LC location. This variability is in accordance with the differences in the location of LC raw data peaks found in the previous study (Keren et al., 2009).

From a conceptual point of view, the individual locations of the LC cannot perfectly be captured by a probability mask surrounding the averaged LC location, and either some relevant signal will be lost or irrelevant signal will be added dependent on the anatomy of the subjects or patients.

Considering that the neuronal cells within the noradrenergic key region LC will be homogeneously working with its known connectivity partners which are directly reached by its strong anatomical projections (Samuels & Szabadi, 2008a; 2008b), we expected that higher accordance of time series within the LC voxels will reflect decreased contamination of the signal by non‐LC voxels.

And indeed, ReHo calculation revealed much greater accordance of the time series after extraction of the signal at the individual LC location. Thus, we interpreted this result as showing a more precise catch of the relevant activation of this nerve cell structure.

Our rs‐fc results are both in line with in vivo fMRI results in humans (Jacobs et al., 2018; Liebe et al., 2018; Song et al., 2017; Wagner et al., 2018; Zhang et al., 2016) and with anatomical and conceptual work regarding LC function (Samuels & Szabadi, 2008a, 2008b). According to Zhang, Song, Wagner and our previous publication, we found strong connections to ACC, bilateral thalamus, and bilateral cerebellar regions, and they were comparable in both MNI and anatomical seed‐based technique.

Because of their dominant connectivity significance in our study, visualization in previous fMRI studies and because of their anatomical and conceptual accordance to LC function the axis LC–thalamus and LC–ACC should be highlighted. These connectivities may represent the baseline activity of attentional control to sensory stimuli:

When exploring brain activity at attentional tasks, three distinct networks can be differentiated: The executive control network, the orienting network and the alerting network (Fan, McCandliss, Fossella, Flombaum, & Posner, 2005; Fan, McCandliss, Sommer, Raz, & Posner, 2002; Xuan et al., 2016).

Within these concepts, ACC and thalamus enhance its activity in tasks requiring executive control as well as thalamic activation is visible in the alerting effect. Executive control of cognition and emotion was predominantly attributed to the ACC (Posner, 2012) which interestingly sends strong projections to the LC (Aston‐Jones & Cohen, 2005b). Furthermore, from an anatomical and conceptual point of view, the involvement of the LC was highlighted in the alerting effect (Petersen & Posner, 2012). Executive control is necessary for resolution of conflict among responses to sensory stimuli (Posner, 2012) and the alerting effect could be interpreted as an enhancement of sensory signal perception after a warning stimulus (Fan et al., 2005). Using the Stroop task, LC was shown to be significantly correlated with the Stroop interference with changing functional connectivity pattern to the dorsal ACC and PFC (Köhler, Bär, & Wagner, 2016), indicating its role in executive control.

We interpret the LC‐thalamic connectivity found here as representing a relay that responds to and manipulate sensory signals that are brought to the thalamus from sensory neurons by bottom‐up connections (Berridge & Waterhouse, 2003; Devilbiss & Waterhouse, 2011; Devilbiss, Waterhouse, Berridge, & Valentino, 2012). Elevation or decline of LC‐thalamic rs‐fc may thus represent differences in baseline response preposition to sensory stimuli within the alerting network. Certain medication can manipulate LC‐thalamic connectivity (Kline et al., 2016; Liebe et al., 2018; Song et al., 2017) and the reported fc change was attributed to attentional functions. Furthermore, in terms of ketamine administration, we interpreted the diminished LC‐thalamic connectivity as relevant for the disturbances in the perception of sensory signals evident in depersonalization and derealization symptoms (Liebe et al., 2018). Future drug studies may keep an eye on LC‐thalamic connectivity and may interpret the results in terms of noradrenergic influence on sensory signal perception, especially if alerting effects play a known role.

LC–ACC connectivity may represent the top‐down control described by Aston‐Jones (Aston‐Jones & Cohen, 2005b). Within the executive control network, sensory signals are evaluated by the ACC in respect to their cost in decision processes. The ACC controls the baseline activity of the LC and consequently the LC‐thalamic relay outlined above. Thus, differences in resting state LC–ACC rs‐fc could represent a reduced or enhanced top‐down control to sensory signal perception and should be carefully assessed in future studies—especially if a noradrenergic component or the involvement of executive control in medication or task studies is expected.

Summing up, the axis ACC–LC–thalamus may play a major role in the guidance of sensory signal perception and can be visualized adequately in 3 T rs fMRI with both MNI space and within subject masks.

The baseline LC‐cerebellar rs‐fc found in this study is in accordance to the results published before and we found no significant differences in comparison of MNI and individual seed method regarding this brain area. The NE spillover in the cerebellum controlled by the LC may influence motor performance (Watson & McElligott, 1984). Methylphenidate, a first‐line ADHD medication, influences LC‐cerebellar fc (Kline et al., 2016), emphasizing the potential of evaluation of LC‐cerebellar connectivity in drug monitoring. Future studies may evaluate LC–cerebellar rs‐fc in respect to motor circuits, but also in terms of emotion and cognition (Schmahmann & Caplan, 2006).

Some regions exhibited unilateral connectivity to LC, such as putamen, amygdala and nucleus accumbens in the MNI space method or left frontal neocortical areas in the individual approach. The connectivity effect sizes of these areas were smaller compared to the brain regions discussed above (for values see Tables 2 and 3), in our interpretation reflecting brain sections that may be less stable connected to LC in the resting situation. These areas are also directly anatomically linked to the LC (Samuels & Szabadi, 2008a; 2008b) and may exhibit connectivity dependent of actual psychological states like inner tension or anxiety (amygdala, McCall et al., 2015), wakefulness (neocortical areas, Berridge, Schmeichel, & España, 2012) or episodic memory retrieval (parahippocampal area, Aminoff, Kveraga, & Bar, 2013).

Finally, we revealed differences in the LC rs‐fc to the right temporoparietal junction (TPJ). The right TPJ is highly involved in the attention networks (Corbetta, Patel, & Shulman, 2008) and furthermore in out of body experiences (Blanke & Arzy, 2005). But since the strong LC‐TPJ connectivity was only visible in the MNI based and not in the subject‐specific approach, we doubt that these connections are predominantly caused by the LC. The LC is surrounded by the central tegmental tract, the medial longitudinal fasciculus and the central gray of the pons (Polak, Kalinin, & Feinstein, 2011), which may contribute to the BOLD signal and to the LC‐TPJ connectivity found here. Thus LC‐TPJ should carefully be interpreted in studies using a MNI space masked approach.

There are some limitations to this study. We cannot exclude small submilimeter shifts in the registration of TSE to T1MPRAGE and in EPI to anatomical coregistration, but in our extensive visual control of preprocessing, we found no relevant displacement in the alignment of these sequences.

Especially automated coregistration of the neuromelanin sensitive sequence to the anatomical sequence was challenging because of the small field of view of the T1TSE sequence. We achieved better results without coregistering T1TSE to the T1MPRAGE images, which was supported by visual inspection.

The LC location was drawn by an experienced neuroradiologist, because exact definition of LC borders by an algorithm is difficult and not reliably established so far. Future studies with higher EPI resolutions may assess inter‐rater reliability to control for the impact of potential differences in LC delineation, which is unlikely to influence the results at the BOLD resolution presented here. In comparison to previous studies we examined a small sample size of 25 men, but could show a reliable and sufficient LC rs‐fc measurement in a relevant group size that may be difficult to exceed in most medicational studies.

## 5 CONCLUSIONS

In high resolution 3 T MRI, the MNI space masks adequately reveal LC rs‐fc to many brain regions involved in attentional processing. Nevertheless, the anatomical within‐subject approach shows a tremendous increase in ReHo, demonstrating higher specificity of LC signal extraction. Furthermore, LC‐TPJ connectivity may be driven by other brainstem nuclei than the LC.

In general, our study underlines the feasibility and relevance of LC rs‐fc measurements in fMRI. We encourage LC rs‐fc measurements in surveys expecting involvement of norepinephrine or attentional networks and provide a basis for the interpretation of forthcoming results. The subject‐specific method may be suitable for future research both at 3 T and especially at higher field strengths which aim to investigate subtle changes in rs‐fc and regional activity of the LC. Since the LC is involved in Alzheimer's disease (Kelly et al., 2017), our method may be interesting for longitudinal studies, where individual variability in the measurements should be reduced to a minimum. The results of this 3 T MRI study are especially relevant for assessment of patient groups, because to date ultrahigh field strengths are not available in a clinical context.

## CONFLICT OF INTEREST

The authors declare no potential conflicts of interest.

## Supporting information


**Data S1** Supporting Information.

## Data Availability

The data that support the findings of this study are available from the corresponding author upon reasonable request.
