# Supplementary material for: In vivo anatomical mapping of human locus coeruleus functional connectivity at 3 T MRI
Source: Hum Brain Mapp. 2020 Jan 28;41(8):2136–51. doi: 10.1002/hbm.24935 (PMC7267980; doi:10.1002/hbm.24935)
Supplement: Supplementary file 1 — Data S1 Supporting Information. [file HBM-41-2136-s001.doc]

**In vivo anatomical mapping of human locus coeruleus functional connectivity at 3T MRI**

**(Supplementary material)**

**Figure 1:** T1 TSE neuromelanine and T1MPRAGE alignment in one exemplary participant. The crossing marks the floor of the fourth ventricle to compare proper fit of the sequences.

**Table 1:** List of ROI‘s included in ROI-to-ROI rs-fc analysis, explanation of ROI abbreviations

Seed based functional connectivity

ROI-to-ROI functional connectivity

Regional homogeneity (ReHo)


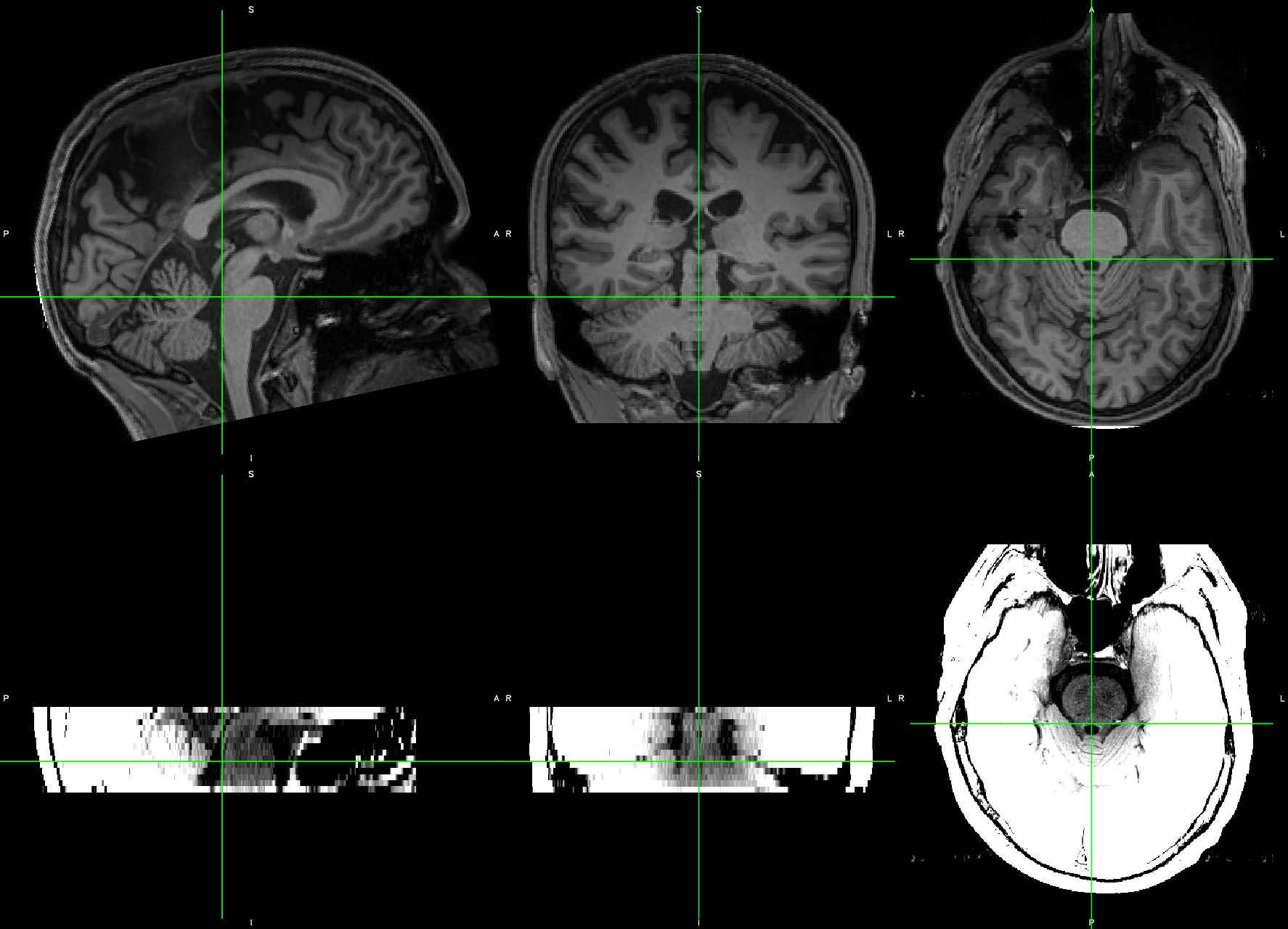


**Figure 1:** T1 TSE neuromelanine and T1MPRAGE alignment in one exemplary participant. The crossing marks the floor of the fourth ventricle to compare proper fit of the sequences.

**Table 1:** List of ROI‘s included in ROI-to-ROI rs-fc analysis, explanation of ROI abbreviations

atlas.FP r (Frontal Pole Right)

atlas.FP l (Frontal Pole Left)

atlas.IC r (Insular Cortex Right)

atlas.IC l (Insular Cortex Left)

atlas.SFG r (Superior Frontal Gyrus Right)

atlas.SFG l (Superior Frontal Gyrus Left)

atlas.MidFG r (Middle Frontal Gyrus Right)

atlas.MidFG l (Middle Frontal Gyrus Left)

atlas.IFG tri r (Inferior Frontal Gyrus, pars triangularis Right)

atlas.IFG tri l (Inferior Frontal Gyrus, pars triangularis Left)

atlas.IFG oper r (Inferior Frontal Gyrus, pars opercularis Right)

atlas.IFG oper l (Inferior Frontal Gyrus, pars opercularis Left)

atlas.PreCG r (Precentral Gyrus Right)

atlas.PreCG l (Precentral Gyrus Left)

atlas.TP r (Temporal Pole Right)

atlas.TP l (Temporal Pole Left)

atlas.aSTG r (Superior Temporal Gyrus, anterior division Right)

atlas.aSTG l (Superior Temporal Gyrus, anterior division Left)

atlas.pSTG r (Superior Temporal Gyrus, posterior division Right)

atlas.pSTG l (Superior Temporal Gyrus, posterior division Left)

atlas.aMTG r (Middle Temporal Gyrus, anterior division Right)

atlas.aMTG l (Middle Temporal Gyrus, anterior division Left)

atlas.pMTG r (Middle Temporal Gyrus, posterior division Right)

atlas.pMTG l (Middle Temporal Gyrus, posterior division Left)

atlas.toMTG r (Middle Temporal Gyrus, temporooccipital part Right)

atlas.toMTG l (Middle Temporal Gyrus, temporooccipital part Left)

atlas.aITG r (Inferior Temporal Gyrus, anterior division Right)

atlas.aITG l (Inferior Temporal Gyrus, anterior division Left)

atlas.pITG r (Inferior Temporal Gyrus, posterior division Right)

atlas.pITG l (Inferior Temporal Gyrus, posterior division Left)

atlas.toITG r (Inferior Temporal Gyrus, temporooccipital part Right)

atlas.toITG l (Inferior Temporal Gyrus, temporooccipital part Left)

atlas.PostCG r (Postcentral Gyrus Right)

atlas.PostCG l (Postcentral Gyrus Left)

atlas.SPL r (Superior Parietal Lobule Right)

atlas.SPL l (Superior Parietal Lobule Left)

atlas.aSMG r (Supramarginal Gyrus, anterior division Right)

atlas.aSMG l (Supramarginal Gyrus, anterior division Left)

atlas.pSMG r (Supramarginal Gyrus, posterior division Right)

atlas.pSMG l (Supramarginal Gyrus, posterior division Left)

atlas.AG r (Angular Gyrus Right)

atlas.AG l (Angular Gyrus Left)

atlas.sLOC r (Lateral Occipital Cortex, superior division Right)

atlas.sLOC l (Lateral Occipital Cortex, superior division Left)

atlas.iLOC r (Lateral Occipital Cortex, inferior division Right)

atlas.iLOC l (Lateral Occipital Cortex, inferior division Left)

atlas.ICC r (Intracalcarine Cortex Right)

atlas.ICC l (Intracalcarine Cortex Left)

atlas.MedFC (Frontal Medial Cortex)

atlas.SMA r (Juxtapositional Lobule Cortex -formerly Supplementary Motor Cortex- Right)

atlas.SMA L(Juxtapositional Lobule Cortex -formerly Supplementary Motor Cortex- Left)

atlas.SubCalC (Subcallosal Cortex)

atlas.PaCiG r (Paracingulate Gyrus Right)

atlas.PaCiG l (Paracingulate Gyrus Left)

atlas.AC (Cingulate Gyrus, anterior division)

atlas.PC (Cingulate Gyrus, posterior division)

atlas.Precuneous (Precuneous Cortex)

atlas.Cuneal r (Cuneal Cortex Right)

atlas.Cuneal l (Cuneal Cortex Left)

atlas.FOrb r (Frontal Orbital Cortex Right)

atlas.FOrb l (Frontal Orbital Cortex Left)

atlas.aPaHC r (Parahippocampal Gyrus, anterior division Right)

atlas.aPaHC l (Parahippocampal Gyrus, anterior division Left)

atlas.pPaHC r (Parahippocampal Gyrus, posterior division Right)

atlas.pPaHC l (Parahippocampal Gyrus, posterior division Left)

atlas.LG r (Lingual Gyrus Right)

atlas.LG l (Lingual Gyrus Left)

atlas.aTFusC r (Temporal Fusiform Cortex, anterior division Right)

atlas.aTFusC l (Temporal Fusiform Cortex, anterior division Left)

atlas.pTFusC r (Temporal Fusiform Cortex, posterior division Right)

atlas.pTFusC l (Temporal Fusiform Cortex, posterior division Left)

atlas.TOFusC r (Temporal Occipital Fusiform Cortex Right)

atlas.TOFusC l (Temporal Occipital Fusiform Cortex Left)

atlas.OFusG r (Occipital Fusiform Gyrus Right)

atlas.OFusG l (Occipital Fusiform Gyrus Left)

atlas.FO r (Frontal Operculum Cortex Right)

atlas.FO l (Frontal Operculum Cortex Left)

atlas.CO r (Central Opercular Cortex Right)

atlas.CO l (Central Opercular Cortex Left)

atlas.PO r (Parietal Operculum Cortex Right)

atlas.PO l (Parietal Operculum Cortex Left)

atlas.PP r (Planum Polare Right)

atlas.PP l (Planum Polare Left)

atlas.HG r (Heschls Gyrus Right)

atlas.HG l (Heschls Gyrus Left)

atlas.PT r (Planum Temporale Right)

atlas.PT l (Planum Temporale Left)

atlas.SCC r (Supracalcarine Cortex Right)

atlas.SCC l (Supracalcarine Cortex Left)

atlas.OP r (Occipital Pole Right)

atlas.OP l (Occipital Pole Left)

atlas.Thalamus r

atlas.Thalamus l

atlas.Caudate r

atlas.Caudate l

atlas.Putamen r

atlas.Putamen l

atlas.Pallidum r

atlas.Pallidum l

atlas.Hippocampus r

atlas.Hippocampus l

atlas.Amygdala r

atlas.Amygdala l

atlas.Accumbens r

atlas.Accumbens l

atlas.Brain-Stem

atlas.Cereb1 l (Cerebelum Crus1 Left)

atlas.Cereb1 r (Cerebelum Crus1 Right)

atlas.Cereb2 l (Cerebelum Crus2 Left)

atlas.Cereb2 r (Cerebelum Crus2 Right)

atlas.Cereb3 l (Cerebelum 3 Left)

atlas.Cereb3 r (Cerebelum 3 Right)

atlas.Cereb45 l (Cerebelum 4 5 Left)

atlas.Cereb45 r (Cerebelum 4 5 Right)

atlas.Cereb6 l (Cerebelum 6 Left)

atlas.Cereb6 r (Cerebelum 6 Right)

atlas.Cereb7 l (Cerebelum 7b Left)

atlas.Cereb7 r (Cerebelum 7b Right)

atlas.Cereb8 l (Cerebelum 8 Left)

atlas.Cereb8 r (Cerebelum 8 Right)

atlas.Cereb9 l (Cerebelum 9 Left)

atlas.Cereb9 r (Cerebelum 9 Right)

atlas.Cereb10 l (Cerebelum 10 Left)

atlas.Cereb10 r (Cerebelum 10 Right)

atlas.Ver12 (Vermis 1 2)

atlas.Ver3 (Vermis 3)

atlas.Ver45 (Vermis 4 5)

atlas.Ver6 (Vermis 6)

atlas.Ver7 (Vermis 7)

atlas.Ver8 (Vermis 8)

atlas.Ver9 (Vermis 9)

atlas.Ver10 (Vermis 10)

networks.DefaultMode.MPFC (1,55,-3)

networks.DefaultMode.LP (L) (-39,-77,33)

networks.DefaultMode.LP (R) (47,-67,29)

networks.DefaultMode.PCC (1,-61,38)

networks.SensoriMotor.Lateral (L) (-55,-12,29)

networks.SensoriMotor.Lateral (R) (56,-10,29)

networks.SensoriMotor.Superior (0,-31,67)

networks.Visual.Medial (2,-79,12)

networks.Visual.Occipital (0,-93,-4)

networks.Visual.Lateral (L) (-37,-79,10)

networks.Visual.Lateral (R) (38,-72,13)

networks.Salience.ACC (0,22,35)

networks.Salience.AInsula (L) (-44,13,1)

networks.Salience.AInsula (R) (47,14,0)

networks.Salience.RPFC (L) (-32,45,27)

networks.Salience.RPFC (R) (32,46,27)

networks.Salience.SMG (L) (-60,-39,31)

networks.Salience.SMG (R) (62,-35,32)

networks.DorsalAttention.FEF (L) (-27,-9,64)

networks.DorsalAttention.FEF (R) (30,-6,64)

networks.DorsalAttention.IPS (L) (-39,-43,52)

networks.DorsalAttention.IPS (R) (39,-42,54)

networks.FrontoParietal.LPFC (L) (-43,33,28)

networks.FrontoParietal.PPC (L) (-46,-58,49)

networks.FrontoParietal.LPFC (R) (41,38,30)

networks.FrontoParietal.PPC (R) (52,-52,45)

networks.Language.IFG (L) (-51,26,2)

networks.Language.IFG (R) (54,28,1)

networks.Language.pSTG (L) (-57,-47,15)

networks.Language.pSTG (R) (59,-42,13)

networks.Cerebellar.Anterior (0,-63,-30)

networks.Cerebellar.Posterior (0,-79,-32)

LC MNI mask

LC individual seed

Seed based functional connectivity

$$Z\left(x\right)=\text{tanh}^{-1}r\left(x\right)$$

with S(x,t): BOLD timeseries at voxel x, R(t): BOLD timeseries within ROI, r(x): correlation coefficients between seed ROI and target voxel, Z(x): Fisher-transformed correlation coefficient (www.nitrc.org/projects/conn RRID:SCR_009550, <https://web.conn-toolbox.org/>, (Whitfield-Gabrieli & Nieto-Castanon, 2012))

ROI-to-ROI functional connectivity

$$Z\left(i,j\right)=\text{tanh}^{-1}r\left(i,j\right)$$

with R_i_(t): BOLD timeseries within i-th ROI, r(i,j): correlation coefficients between i-th and j-th ROIs, Z(i,j): Fisher-transformed correlation coefficient

(www.nitrc.org/projects/conn RRID:SCR_009550, <https://web.conn-toolbox.org/>, (Whitfield-Gabrieli & Nieto-Castanon, 2012))

Regional homogeneity (ReHo)

with *W*=Kendalls‘s W, *K*: number of neighboring voxels (27), *R_i_*: sum of rank of all K voxels at frame i, *n*: length of time series,$\bar{R}$: average R_i_ across frames (as calculated in BRANT software (Xu, Liu, Zhan, Ren, & Jiang, 2018))
